# COPD and cognitive impairment: a review of associated factors and intervention strategies

**DOI:** 10.3389/fpsyt.2025.1714708

**Published:** 2026-01-19

**Authors:** Yuling Jing, Shuixiang Mao

**Affiliations:** Department of Nursing, Mianzhu People’s Hospital, Deyang, Sichuan, China

**Keywords:** COPD, cognitive impairment, associated factors, intervention strategies, systemic inflammation

## Abstract

Chronic obstructive pulmonary disease (COPD) is increasingly recognized as a systemic disorder associated with heightened risk of cognitive impairment, including mild cognitive impairment (MCI) and dementia. Epidemiological studies indicate COPD patients face a 1.74-fold higher risk of cognitive decline, with deficits predominantly affecting attention, memory, and executive functions, impairing daily living and increasing mortality risk. This review synthesizes factors linking COPD to cognitive impairment, including systemic inflammation (via proinflammatory cytokines and blood-brain barrier disruption), hypoxemia/hypercapnia (inducing oxidative stress and neuronal damage), acute exacerbations (exacerbating inflammation and persisting deficits), and comorbidities like obstructive sleep apnoea (OSA), cerebral microbleeds, and depression. Smoking’s role remains paradoxical, with neurotoxicants potentially counteracted by nicotine’s neuroprotective effects. Assessment relies on neuropsychological tools (e.g., MoCA, MMSE), neurophysiological measures (P300 ERP), and neuroimaging, though limitations persist. Interventions focus on non-pharmacological strategies: pulmonary rehabilitation (improving cognition via enhanced cerebral perfusion), cognitive training (targeting memory/attention), and long-term oxygen therapy (LTOT, reducing decline in hypoxemic patients). Critical gaps include unclear mechanisms and the need for personalized interventions. Addressing these may improve clinical outcomes and quality of life in COPD patients.

## Introduction

Chronic obstructive pulmonary disease (COPD) is a preventable and treatable respiratory disorder characterized by persistent, progressive airflow limitation. Beyond its primary pulmonary manifestations, COPD is increasingly recognized as a systemic disease associated with a spectrum of extrapulmonary comorbidities, including ischemic heart disease, hypertension, and metabolic syndrome. Among these, cognitive impairment has emerged as a significant and underappreciated extrapulmonary complication, drawing growing attention in recent clinical and research endeavors (1).

Epidemiological evidence consistently demonstrates that individuals with COPD face a substantially elevated risk of cognitive impairment compared to those without the disease (2). A longitudinal study involving 9,765 community-dwelling older adults in China revealed that the incidence of cognitive impairment over a 3-year follow-up was 21.7% in COPD patients, significantly higher than the 16.2% observed in their non-COPD counterparts (3). Furthermore, after adjusting for confounding variables such as age and educational attainment, COPD patients were found to have a 1.74-fold increased risk of developing cognitive impairment (4). The cognitive domains most frequently affected in COPD include attention, memory, and executive functions, which are pivotal for maintaining independent daily living.

In COPD, cognitive impairment primarily affects the domains of attention, memory, and executive function (5). Studies show that attention deficits are particularly prevalent in COPD patients, leading to difficulties in focusing and sustaining attention during daily activities. Memory problems, especially in working memory and episodic memory, are commonly observed, which significantly impact the ability to recall important information and perform tasks that require remembering sequences or details (6). Additionally, executive function, including skills like planning, problem-solving, and decision-making, is often impaired, particularly in more severe stages of COPD. These cognitive deficits, especially in attention, memory, and executive function, are associated with poorer functional outcomes, including increased dependence on others and a lower quality of life (7). In contrast, dementia is a syndrome marked by progressive, acquired cognitive decline that severely compromises daily living, learning, and social interaction capacities. Alzheimer’s disease (AD) remains the most extensively studied subtype of dementia (8).

The occurrence of cognitive impairment in COPD patients has profound clinical implications, including reduced self-care capacity, diminished quality of life, and increased risks of hospitalization and mortality (9). Given these adverse outcomes, early identification and assessment of cognitive function in COPD patients are of paramount importance to mitigate the risk of cognitive decline (10). However, the underlying mechanisms linking COPD to cognitive impairment remain incompletely elucidated (2). Although several reviews have explored the relationship between COPD and cognitive impairment, this review provides a more comprehensive analysis, integrating the latest research findings to delve into the multifactorial pathophysiological mechanisms of cognitive impairment in COPD patients. Furthermore, this review places particular emphasis on the application of non-pharmacological intervention strategies and offers specific recommendations that incorporate nursing practice, aiming to fill the gaps in the existing literature. This mini-review aims to synthesize current knowledge on the epidemiology, associated factors, and intervention strategies for cognitive impairment in COPD, with the goal of providing insights to inform clinical practice and improve patient outcomes.

## Methods

This review systematically examined literature on cognitive impairment in Chronic Obstructive Pulmonary Disease (COPD). We included peer-reviewed studies published in English that focused on the relationship between COPD and cognitive decline, particularly mild cognitive impairment (MCI) and dementia. A comprehensive literature search was conducted in PubMed, Scopus, and Web of Science using keywords such as “COPD,” “cognitive impairment,” “mild cognitive impairment,” “dementia,” “systemic inflammation,” and “interventions.

Inclusion criteria were (1): studies on adults aged 40+ with diagnosed COPD (2), studies investigating the impact of COPD on cognitive function, and (3) studies addressing both pathophysiological mechanisms and non-pharmacological interventions. Studies were excluded if they (1) did not focus on cognitive outcomes in COPD, (2) were not peer-reviewed, or (3) were not original research (e.g., reviews, editorials).

Eligible studies were assessed for relevance, quality, and methodology. Data were synthesized to provide an overview of the current state of research on COPD-related cognitive impairment, including contributing factors, assessment methods, and intervention strategies.

### Factors associated with cognitive impairment in COPD patients

Cognitive impairment in COPD arises from a complex interplay of multiple factors, including inflammation, hypoxemia, hypercapnia, acute exacerbations, comorbidities (obstructive sleep apnoea, cerebral microbleeds, anxiety, and depression), and smoking (11).

### Inflammation

Chronic pulmonary inflammation in COPD disrupts the balance between immune system-mediated damage and repair mechanisms (12). Inflammatory mediators from the airways enter systemic circulation, triggering a systemic inflammatory response that compromises the structure and function of extrapulmonary organs (13). This systemic inflammation contributes to the pathogenesis of cognitive impairment through mechanisms such as microglial activation, elevated levels of proinflammatory cytokines, complement cascade activation, and blood-brain barrier disruption—processes also implicated in the development of AD (14, 15). Activated microglia release a spectrum of proinflammatory mediators, including neutrophils, macrophages, interleukin-6 (IL-6), interleukin-8 (IL-8), tumour necrosis factor-α (TNF-α), and C-reactive protein (CRP), which collectively impair cognitive function (11). In stable COPD patients, elevated CRP levels—attributed to chronic inflammation—have been associated with cognitive decline, though no significant correlations between IL-6, IL-8, TNF-α, and cognitive performance have been identified. Long-term inflammatory stimulation damages vascular endothelium, disrupts blood-brain barrier integrity, and alters cerebrovascular structure, leading to impairments in attention and memory (16). These findings underscore inflammation as a key driver of cognitive impairment in COPD through multifaceted pathological pathways (17).

### Hypoxemia and hypercapnia

Alveolar hypoventilation in COPD frequently results in hypoxemia and hypercapnia, both of which contribute to cognitive decline (18). Hypoxia induces oxidative stress and inflammatory responses, directly damaging neurons and reducing the synthesis and release of neurotransmitters involved in acetylcholine metabolism—critical for cognitive processes such as memory and attention (19). Sustained hypoxia also reduces cerebral perfusion, diminishes neuronal activity, and promotes neuronal apoptosis (20). Additionally, hypoxia-driven overproduction of reactive oxygen species exacerbates neuronal injury, further impairing cognitive function. A longitudinal study demonstrated that COPD patients with oxygen saturation ≤88% face a higher risk of cognitive impairment, highlighting the dose-dependent relationship between hypoxemia and cognitive decline (21).

Hypercapnia, often severe in advanced COPD (as indicated by higher GOLD stages), exerts deleterious effects on the central nervous system. Elevated carbon dioxide levels cause cerebral vasodilation, increasing cerebral blood volume and intracranial pressure, which exacerbates brain injury (22). Acidosis (secondary to hypercapnia) increases vascular permeability, leading to cerebral edema, while elevated cerebrospinal fluid H+ concentrations disrupt cellular metabolism and suppress cortical activity (20). Concomitant acidosis, edema, and hypoxia enhance glutamate decarboxylase activity in neurons, further impairing cellular function. Functional connectivity—assessed via resting-state functional magnetic resonance imaging— is compromised in hypercapnic patients, representing a potential neurobiological substrate for cognitive decline. Moreover, the severity of carbon dioxide retention correlates with memory impairment, solidifying hypercapnia as an independent risk factor for cognitive dysfunction in COPD (22).

### Acute exacerbations of COPD

Acute exacerbations of COPD—defined as acute worsening of respiratory symptoms beyond daily variability, requiring treatment adjustments—significantly impact cognitive function. The severity of COPD correlates with cognitive performance, with exacerbations mediating cognitive decline through heightened inflammation and reduced pulmonary function (23). Inflammatory markers inversely correlate with cognitive scores during exacerbations. Pulmonary function indices, particularly forced vital capacity (FVC), decline with increasing airflow limitation and strongly associate with cognitive impairment, whereas forced expiratory volume in 1 second (FEV_1_) shows no consistent relationship (5). A 3-month cohort study revealed that cognitive function is significantly poorer during exacerbations compared to stable phases, with deficits persisting even after clinical recovery (21). After adjusting for age, education, and smoking, frequent exacerbations independently predict cognitive decline, with higher exacerbation frequency correlating with more severe cognitive impairment, positioning exacerbation history as a prognostic indicator for cognitive outcomes in COPD.

### Obstructive sleep apnoea

OSA is highly prevalent in COPD, with reported comorbidity rates ranging from 45% to 65.9% in clinical cohorts. OSA-induced recurrent nocturnal hypoxia and sleep fragmentation directly impair cognitive function, particularly memory (24). Sleep architecture disruption in OSA promotes pathological accumulation of cerebrospinal fluid amyloid-β (Aβ) and tau proteins—hallmarks of AD—while increasing central nervous system oxidative stress and blood-brain barrier permeability. Cross-sectional studies demonstrate that COPD patients with comorbid OSA exhibit a higher risk and greater severity of cognitive impairment compared to those without OSA (25). Both OSA and COPD independently impair attention, memory, executive function, psychomotor speed, and language, with additive effects observed in comorbid patients, underscoring OSA as a critical modifiable risk factor for cognitive decline in COPD.

### Cerebral microbleeds

CMBs—small, hemosiderin-laden lesions resulting from microvascular leakage—are a common cerebral small vessel disease in COPD. Longitudinal data indicate that 45% of COPD patients develop CMBs, compared to 31% of non-COPD individuals, with risk increasing alongside airflow limitation and dyspnea severity (16). CMBs disrupt multiple cognitive domains, including orientation, attention, calculation, and delayed recall, with the severity of cognitive impairment inversely correlating with lesion burden. The anatomical distribution of CMBs further modulates cognitive outcomes, with region-specific effects on memory and executive function (26). These findings establish CMBs as a key neurovascular mediator of cognitive decline in COPD.

### Depression and anxiety

**Figure 1:**
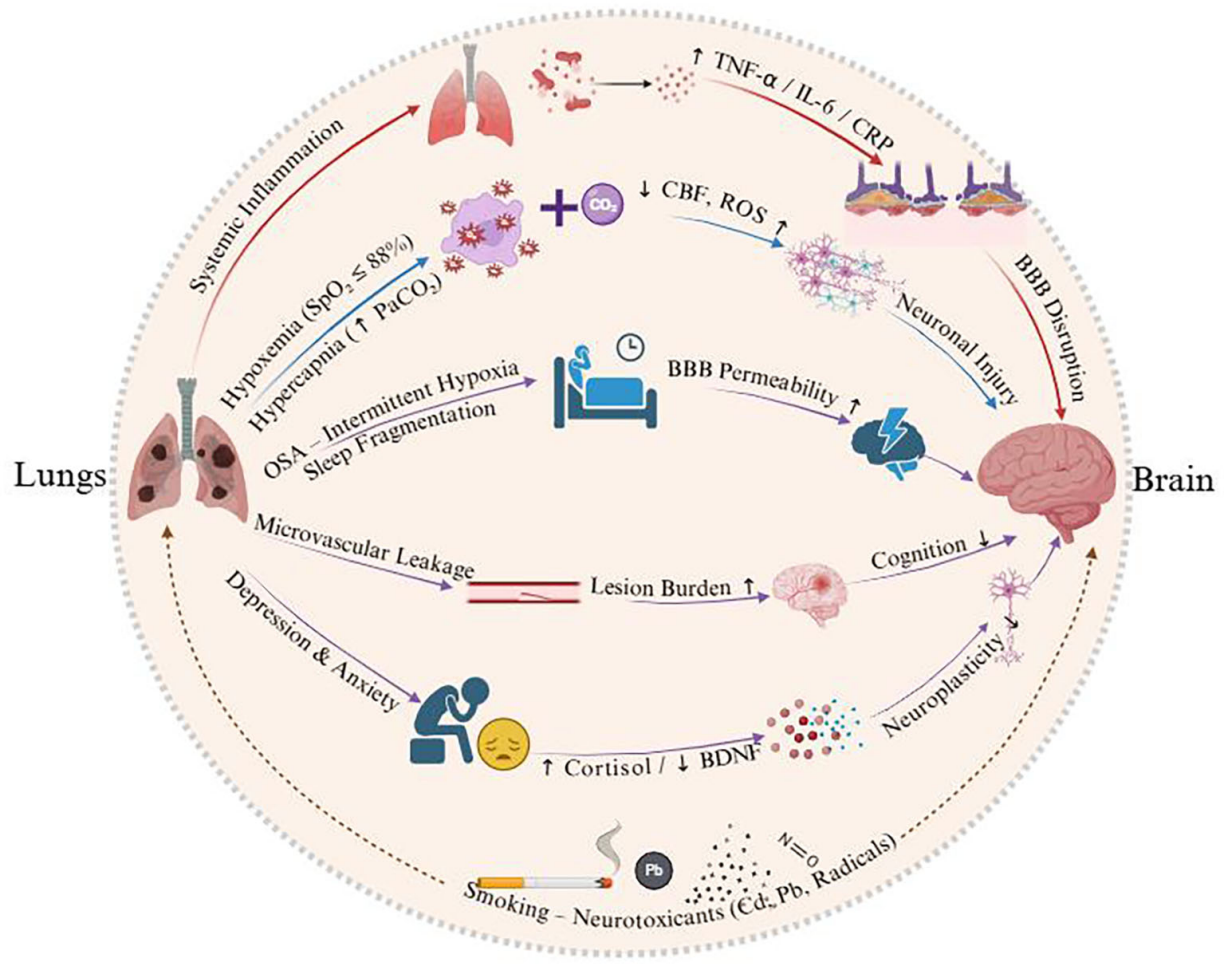
Schematic illustrating the pathways linking COPD to cognitive impairment via pulmonary dysfunction, systemic inflammation, and comorbid factors.

Over one-third of COPD patients experience comorbid depression and anxiety, which exacerbate cognitive impairment. Chronic respiratory dysfunction, physical inactivity, and social isolation in COPD contribute to negative affect, while anxiety-induced elevations in cortisol and reductions in brain-derived neurotrophic factor directly impair neuronal plasticity and cognitive performance (27). Epidemiological data show a stepwise increase in MCI prevalence with increasing depression severity: 10.0% in non-depressed patients, 13.3% in mild depression, and 19.7% in moderate-to-severe depression (28). Depressed COPD patients face a higher risk of cognitive impairment compared to their non-depressed counterparts, highlighting the bidirectional relationship between mood disorders and cognitive decline in COPD (29). **Figure 1** illustrates the pathway by which COPD is linked to cognitive impairment through pulmonary dysfunction, systemic inflammation, and comorbid factors.

### Smoking

Smoking is a dual risk factor for COPD and cognitive impairment, though its net effect remains contentious. Cigarette smoke contains neurotoxicants such as cadmium, nitric oxide, lead, and free radicals, which alter gray and white matter volumes and impair cognitive function. Observational studies identify smoking as a risk factor for cognitive impairment in COPD (30). Although nicotine—a constituent of tobacco—has been hypothesized to exert short-term neuroprotective effects by enhancing acetylcholine release and transiently improving attention or information processing (31), current large-scale cohort studies and meta-analyses indicate that these potential benefits are outweighed by the substantial neurovascular and oxidative harms of chronic smoking. Moreover, smoking cessation, rather than reduction, is associated with improved cognitive outcomes and reduced dementia risk in the general population and COPD cohorts (32). Therefore, the overall evidence supports that the detrimental effects of smoking on cognition far outweigh any transient or theoretical neuroprotective benefits of nicotine, underscoring the importance of smoking cessation as a critical preventive strategy for cognitive decline in COPD patients (33) (**Table 1**).

**Table 1:** Major factors associated with cognitive impairment in COPD.

Factor category	Specific factors	Pathological mechanisms	Key evidence	References
Inflammation	Systemic inflammation, proinflammatory cytokines (IL-6, IL-8, TNF-α, CRP)	Chronic pulmonary inflammation triggers systemic immune activation; microglial activation releases proinflammatory mediators; complement cascade activation and blood-brain barrier disruption.	Elevated CRP in stable COPD correlates with cognitive decline; vascular endothelial damage impairs attention and memory.	(11–13, 16)
Hypoxia & Hypercapnia	Hypoxemia (SpO_2_ ≤ 88%), hypercapnia	Hypoxia: Oxidative stress, reduced cerebral perfusion, neuronal apoptosis; hypercapnia: Cerebral vasodilation, acidosis-induced edema, disrupted metabolism.	SpO_2_ ≤ 88% increases cognitive impairment risk; CO_2_ retention correlates with memory decline; hypercapnia reduces brain functional connectivity.	(20–22)
COPD Exacerbations	Acute exacerbations, frequency, reduced FVC	Exacerbations exacerbate inflammation; FVC decline links to cognitive impairment; deficits persist post-recovery; frequent exacerbations predict worse function.	Exacerbation phase cognitive function is poorer than stable phase; frequency predicts decline.	(5, 23)
Comorbidities	Obstructive Sleep Apnoea (OSA)	OSA (45-65.9% in COPD) causes recurrent hypoxia and sleep fragmentation; promotes Aβ and tau accumulation.	COPD+OSA patients have higher cognitive impairment risk and severity vs. COPD alone.	(24, 25)
	Cerebral Microbleeds (CMBs)	CMBs (45% in COPD vs. 31% in non-COPD) from microvascular leakage; risk increases with airflow limitation; disrupt multiple cognitive domains.	CMBs associate with cognitive decline; lesion location modulates severity.	(16, 26)
	Depression & Anxiety	Over 1/3 of COPD patients affected; anxiety increases cortisol, reduces neurotrophic factors; depression raises cognitive impairment risk.	MCI prevalence higher with worse depression; depressed COPD patients have higher cognitive impairment risk.	(27, 28)
Smoking	Cigarette smoke components, nicotine	Neurotoxicants cause gray/white matter abnormalities; nicotine may enhance acetylcholine release (controversial).	Smoking is a risk factor for cognitive impairment in COPD; role in AD is debated.	(30, 31)

### Pharmacotherapy-Related Factors

Medications used in COPD management may contribute to cognitive decline, as high anticholinergic burden is associated with an increased risk of dementia in older adults (34). Systemic corticosteroid use has also been linked to memory impairment and other cognitive deficits (35). Careful prescription using the lowest effective dose, shortest duration, and monitoring cumulative burden may help mitigate these risks.

### Assessment methods for MCI in COPD

The accurate evaluation of cognitive function in COPD patients with MCI is critical, given that their cognitive profiles may differ from those with age-related MCI (36). A comprehensive understanding of available assessment tools—encompassing neuropsychological, neurophysiological, and neuroimaging approaches—is essential to guide clinical practice and research.

### Neuropsychological assessment

Neuropsychological tests, primarily relying on validated scales, are valued for their simplicity and ease of data processing. They are categorized into two main types:

1. Global cognitive function assessments: The Mini-Mental State Examination (MMSE) and Montreal Cognitive Assessment (MoCA) are the most widely used. The MMSE, available in multiple language versions, remains a cornerstone for cognitive screening in clinical, research, and community settings due to its broad applicability (37). The MoCA, however, demonstrates superior sensitivity and specificity for detecting MCI, effectively distinguishing mild cognitive decline from normal age-related memory changes. For clinical interpretation, a score below 24/30 on the MMSE typically suggests cognitive impairment. The MoCA demonstrates superior sensitivity for detecting MCI, with a standard cut-off score of <26/30; one point is added for individuals with ≤12 years of education to correct for bias. Its enhanced ability to identify subtle deficits in executive function and visuospatial skills makes it more robust than the MMSE for early MCI detection (38).
2. Domain-specific assessments: These target discrete cognitive domains frequently impaired in COPD:

1. Attention: Tests such as the Digit Span Test (simple and widely used) consistently reveal lower scores in COPD patients compared to age-matched controls, indicating attention deficits (39).
2. Memory: The Wechsler Memory Scale (40), Rivermead Behavioral Memory Test (41), and Clinical Memory Scale assess memory function. The Rivermead scale is particularly useful for evaluating episodic memory and tracking changes in memory performance before and after interventions.
3. Executive function: The Trail Making Test and Wisconsin Card Sorting Test are validated tools. The Trail Making Test, with strong construct validity and test-retest reliability, is commonly employed for early screening of MCI in COPD due to its sensitivity to executive dysfunction (42).

### Neurophysiological assessment

Neurophysiological methods offer objective, sensitive measures of cognitive function, with event-related potentials (ERPs) and evoked potentials being the primary modalities. The auditory ERP component P300 has emerged as a valuable adjunct in early MCI diagnosis (43). Its latency correlates with arterial oxygen tension, oxygen saturation, pulmonary function severity, and age, reflecting the interplay between systemic and cognitive impairment in COPD. While both MMSE and MoCA are useful for cognitive screening, previous studies generally suggest that MoCA shows higher sensitivity than MMSE in detecting mild cognitive impairment, including in COPD patients (44).

### Neuroimaging assessment

Neuroimaging techniques, including structural and functional magnetic resonance imaging (MRI), diffusion tensor imaging, and magnetic resonance spectroscopy, provide objective, reproducible data to support early MCI detection, diagnosis, and mechanistic research. Structural MRI reveals progressive brain atrophy and white matter tract disruption with advancing COPD severity, while functional MRI identifies alterations in default mode network (DMN) connectivity—key neural substrates of cognitive function (42).

However, limitations exist: some COPD patients with overt cognitive deficits (documented via neuropsychological tests) show minimal changes in gray or white matter volume, particularly in the absence of hypoxemia or hypercapnia (45). This dissociation underscores the inability of current neuroimaging modalities to fully capture the neurobiological basis of cognitive impairment in COPD, emphasizing the need for multimodal assessment strategies.

### Other assessment and evaluation methods

In addition to the neuropsychological and neurophysiological methods described above, other cognitive assessment tools and biomarkers are gaining attention in predicting the risk of MCI or dementia in COPD patients. For example, tools like the Clock Drawing Test (CDT) and Addenbrooke’s Cognitive Examination (ACE) are sensitive to subtle cognitive deficits in other populations, though studies in COPD patients are limited (46). These tools may require adaptation for COPD-specific cognitive profiles, including adjustments for education and COPD-related pathologies (e.g., hypoxemia, inflammation). Emerging biomarkers, such as amyloid-beta, tau imaging, and inflammatory markers like CRP and IL-6, may provide insight into cognitive decline mechanisms in COPD, but further research is needed to confirm their predictive value for MCI and dementia in COPD (47, 48). Digital health tools, such as CANTAB and wearable devices monitoring cognitive and physiological data, could offer real-time monitoring for early cognitive decline detection (49). These tools are still being evaluated but may become valuable for longitudinal tracking of cognitive changes in COPD. Given these promising directions, further large-scale, multi-center studies are needed to validate these tools’ effectiveness in predicting cognitive decline in COPD (**Table 2**).

**Table 2:** Assessment methods for mild cognitive impairment in COPD.

Assessment category	Specific tools	Key characteristics	Advantages	Limitations	References
Neuropsychological Assessment	Global function: MMSE, MoCA Domain-specific: - Attention: Digit Span Test - Memory: Rivermead Behavioral Memory Test, Wechsler Memory Scale - Executive function: Trail Making Test, Wisconsin Card Sorting Test	Relies on validated scales; evaluates global and discrete cognitive domains (attention, memory, executive function). MoCA is more sensitive to MCI than MMSE.	Simple to administer; easy data processing; MoCA effectively distinguishes MCI from normal aging.	MMSE less sensitive to subtle deficits; domain-specific tests may require training for accurate administration.	(37, 38)
Neurophysiological Assessment	Event-related potential (ERP): Auditory P300	Objective, sensitive measure; P300 latency correlates with arterial oxygen tension, SpO_2_, pulmonary function, and age.	Comparable to MoCA in MCI prevalence screening; reflects physiological links to COPD severity.	Less sensitive than MMSE in non-hypoxemic elderly COPD patients.	(43)
Neuroimaging Assessment	Structural MRI, functional MRI (fMRI), diffusion tensor imaging (DTI), magnetic resonance spectroscopy (MRS)	Detects brain structural changes (atrophy, white matter disruption) and functional alterations (default mode network [DMN] dysfunction) with COPD progression.	Objective, reproducible; aids early detection, diagnosis, and mechanistic research.	Fails to detect significant gray/white matter changes in some COPD patients with neuropsychologically confirmed cognitive decline (especially without hypoxemia/hypercapnia).	(42)

### Intervention strategies for cognitive impairment in COPD

To date, no pharmacological agents have been specifically approved for treating cognitive impairment in COPD, making non-pharmacological interventions the mainstay of management. These strategies primarily include pulmonary rehabilitation, cognitive training, and long-term oxygen therapy (LTOT), each with distinct mechanisms and clinical implications.

### Pulmonary rehabilitation

Pulmonary rehabilitation is a comprehensive, individualized intervention program developed following a thorough patient assessment, encompassing respiratory training, airway clearance techniques, and exercise training—with exercise as its core component (24). Mechanistic studies suggest that exercise exerts neuroprotective effects by increasing cerebral blood flow, upregulating brain-derived neurotrophic factor, and mitigating hippocampal atrophy, thereby reducing the risk of cognitive decline (50).

Clinical evidence supports its efficacy: combined aerobic and resistance training has been shown to significantly improve cognitive domains such as intelligence, attention, and reasoning in older COPD patients. A structured 8-week pulmonary rehabilitation program (3 sessions/week), incorporating respiratory exercises, muscle strengthening, endurance training, self-management education, and nutritional support when needed, not only acutely improves cognitive function in COPD patients with MCI but also maintains these benefits for up to 3 months post-intervention. However, the precise mechanisms linking exercise to cognitive enhancement in COPD remain incompletely understood, partly due to confounding variables such as individual health status and exercise adherence, and the paucity of studies focusing specifically on COPD rather than healthy aging populations (51).

### Cognitive training

Cognitive training aims to enhance cognitive function by improving cerebral perfusion and promoting functional brain reorganization. Multidimensional training protocols—including puzzle-solving, image recognition and recall, and verbal association tasks—have demonstrated efficacy: a 12-week program (1 session/week) significantly improves global cognitive function, memory, attention, and spatial orientation in MCI patients (52). Additionally, combining cognitive training with neuroprotective agents (e.g., herbal extracts) has shown synergistic benefits in improving cognitive performance in COPD patients with cognitive impairment (53).

Challenges persist, however: long-term adherence is required for sustained benefits, and elderly individuals with lower educational attainment may face barriers in understanding and engaging with training protocols, necessitating tailored approaches to optimize compliance.

### Long-term oxygen therapy

LTOT, defined as daily oxygen supplementation for ≥15 hours in patients with chronic hypoxemia, is a cornerstone of COPD management, with established benefits in improving survival. Its role in preserving cognitive function is increasingly recognized: LTOT reduces the risk of cognitive impairment in hypoxemic COPD patients. A randomized controlled trial comparing 45 COPD patients with and without LTOT demonstrated significantly worse cognitive outcomes in the non-oxygenated group, confirming a protective effect (54). Mechanistically, while LTOT does not alter cerebral blood flow, it enhances cerebral oxygen delivery and neurovascular function—key pathways linking oxygen supplementation to reduced risks of stroke, MCI, and dementia.

Despite its efficacy, LTOT is limited by high economic costs, poor patient adherence, and challenges in personalizing oxygen delivery protocols, highlighting the need for strategies to optimize its implementation in clinical practice.

### Nursing role in non-pharmacological interventions

Nurses are essential in implementing non-pharmacological interventions for cognitive impairment in COPD patients. Pulmonary rehabilitation, for example, often requires nursing support in educating patients about the benefits of physical activity and monitoring exercise regimens (55). Nurses can provide tailored guidance based on the patient’s physical and cognitive abilities (56). Cognitive training programs, designed to target memory and attention, can be integrated into routine nursing care, with nurses offering structured sessions and feedback (57). Nurses also assist in managing LTOT by ensuring proper adherence, adjusting oxygen levels as needed, and providing emotional support to patients (58). In addition, nurses integrate behavioral and psychosocial interventions, such as motivational interviewing, psychoeducation for emotional symptoms, and managing treatment plans. Involving caregivers and family members in the patient’s care process improves the effectiveness and long-term success of these interventions. The nursing team plays an integral role in enhancing self-management skills and maintaining patient motivation, ensuring that these interventions are both effective and sustainable (**Table 3**).

**Table 3:** Non-pharmacological intervention strategies for cognitive impairment in COPD: core components, mechanisms, and limitations.

Intervention type	Pulmonary rehabilitation	Cognitive training	Long-term oxygen therapy (LTOT)
Core Components	Comprehensive program including respiratory training, airway clearance, exercise training (aerobic + resistance training as core), muscle strengthening, endurance training, self-management education, and nutritional support when needed.	Multidimensional protocols: puzzle-solving, image recognition/recall, verbal association tasks; may be combined with neuroprotective agents.	Daily oxygen supplementation for ≥15 hours in patients with chronic hypoxemia.
Mechanisms of Action	Increases cerebral blood flow; upregulates brain-derived neurotrophic factor; mitigates hippocampal atrophy.	Improves cerebral perfusion; promotes functional brain reorganization.	Enhances cerebral oxygen delivery and neurovascular function (no effect on cerebral blood flow).
Limitations/Challenges	Mechanisms not fully clarified; influenced by individual health status and exercise adherence; most studies focus on healthy aging rather than COPD specifically.	Requires long-term adherence; elderly with lower educational attainment may face difficulties in understanding and engaging with training.	High economic costs; poor patient adherence; challenges in personalizing oxygen delivery protocols.
References	(50, 51)	(52, 53)	(54)

### CPAP therapy for OSA in COPD patients

For COPD patients with comorbid obstructive sleep apnea (OSA) who do not require long-term oxygen therapy (LTOT), Continuous Positive Airway Pressure (CPAP) therapy has proven beneficial in reducing cognitive impairment (59). CPAP treatment, especially when used for over 4 hours per night, alleviates the intermittent hypoxia and sleep fragmentation characteristic of OSA (60). This has been shown to significantly improve cognitive performance, particularly in domains such as memory, attention, and executive function. Studies have demonstrated that CPAP can enhance the quality of life by improving sleep quality, reducing daytime sleepiness, and protecting against cognitive decline, even in patients with mild cognitive impairment (MCI) or dementia associated with OSA (61).

## Conclusion

COPD is strongly associated with an elevated risk of cognitive impairment, encompassing MCI and, in severe cases, progression to dementia. This relationship is driven by a complex interplay of pathological factors: systemic inflammation—mediated by proinflammatory cytokines, microglial activation, and blood-brain barrier disruption—contributes to neurocognitive decline, while hypoxemia and hypercapnia induce oxidative stress, neuronal damage, and functional brain connectivity deficits. Acute exacerbations of COPD further exacerbate cognitive impairment, with frequent exacerbations serving as a reliable predictor of poorer cognitive outcomes, likely through amplified inflammation and reduced pulmonary function (notably forced vital capacity). Comorbidities such as obstructive sleep apnoea (OSA), cerebral microbleeds, and mood disorders (anxiety and depression) synergistically increase cognitive risk, while smoking exerts dual, poorly understood effects—neurotoxicity via heavy metals and free radicals versus potential neuroprotective properties of nicotine.

Current interventions for cognitive impairment in COPD focus on non-pharmacological strategies. Pulmonary rehabilitation, incorporating aerobic and resistance training, enhances cognitive domains such as attention and executive function through increased cerebral perfusion and neurotrophic factor upregulation, with benefits sustained post-intervention. Cognitive training, via structured tasks targeting memory and reasoning, promotes functional brain reorganization, though long-term adherence and accessibility for elderly patients with limited education remain challenges. LTOT reduces cognitive decline risk in hypoxemic patients by improving cerebral oxygen delivery and neurovascular function, despite barriers of cost and adherence.

Nursing care plays a pivotal role in the management of cognitive impairment in COPD patients. Nurses are at the forefront of monitoring cognitive function, using tools such as the MoCA and MMSE for early detection. They are also critical in implementing non-pharmacological interventions, including pulmonary rehabilitation, cognitive training, and ensuring proper adherence to LTOT. Through their ongoing interaction with patients, nurses provide essential education, emotional support, and motivation, which are key to enhancing self-management and sustaining the benefits of these interventions.

Although several studies have explored the relationship between COPD and cognitive impairment, there are some limitations in the existing research. Some studies have small sample sizes and fail to account for multiple comorbidities, affecting the generalizability and reliability of the results. Additionally, most studies focus on individual factors, such as hypoxemia or inflammation, without a comprehensive analysis of how these factors interact. And the precise mechanisms linking COPD to cognitive impairment require clarification, and optimized, personalized interventions—addressing variability in patient adherence, comorbidity profiles, and disease severity—are needed. Further research into the neurobiological substrates of COPD-related cognitive decline and the development of targeted, scalable interventions will be pivotal to mitigating cognitive risk, enhancing self-care capacity, and reducing the disease burden for COPD patients. Additionally, further nursing research is needed to explore tailored interventions that can improve cognitive outcomes, enhance self-management, and ultimately improve quality of life for COPD patients.
